# The Revised Pension Arrangements, and a New Act

**Published:** 1917-06-16

**Authors:** 


### NATIONAL INSURANCE ACT DEVELOPMENTS.

### The Revised Pension Arrangements, and a New Act.

Another National Insurance Act received the Royal Assent, and became law on May 17 last. Its full title is "National Insurance (Part I. Amendment) Act, 1917—An Act to amend the Enactments relating to National Health Insurance with respect to persons suffering from disablement in consequence of the present war." It contains only four operative sections, but it is nevertheless of considerable importance as affecting the interests of sailors and soldiers who were insured under the National Health Insurance Acts, and who have been granted pensions or allowances by the naval or military authorities on account of disablement in the present war.

The full significance of the measure is not readily apparent, and it was for this reason that considerable delay occurred in its passage through Parliament. Its real purport and effect were, indeed, so little understood that, instead of it passing through all its stages in a single day, as the Government had expected, it was given a second reading only on condition that a full explanation should be furnished at the committee stage, and even here further trouble ensued which necessitated renewed delay. The principal difficulty arose by reason of the fact that the chief section is an amendment of the Amendment Act of 1915, and legislation by reference to sections of earlier Acts does not lend itself to comprehension except to the legally trained mind.

In these circumstances we may, perhaps, be allowed to give a short account of its provisions in such a manner as to make them readily intelligible.

### Why the New Act was Wanted.

It became apparent at a very early stage in the war that, as the contingency of a great European war had not entered into the calculations upon which the finance of the National Insurance scheme was based, it would be impossible for National Insurance funds to bear the strain that would be placed upon them by reason of the disablements suffered by so many insured men while serving with His Majesty's Forces. Concurrently, it became a matter upon which the opinion of the nation was unanimous that the compensation, such as it was, that could be given in these cases of injury and disablement, should rightly fall to be discharged by the nation as a whole—that is to say, the cost should be provided out of Parliamentary grants. To give effect to these principles, the National Insurance Amendment Act of 1915 was duly entered upon the Statute-book. Experience had shown, however, that in its working it had largely failed to effect its object. The reason was that the Act provided that relief should be afforded to National Health Insurance Funds to the extent of 5s. a week, where a pension had been granted in respect of "total disablement," but no provision was made with regard to similar relief if the pension was in the nature of a partial disability allowance. In point of fact, it was seldom that a "total disablement" pension was granted, for, although it might well happen that the sailor or soldier was totally incapacitated and unable to do any work at the time the pension was granted, yet in due course he might—and, doubtless, in the majority of cases would—recover his ability to do some work, though not necessarily that to which he had been accustomed prior to joining the Forces. It was in order to preserve the right of reviewing the pension from time to time that the grant was made in the form of a partial disability allowance rather than under the title of "total disablement."

Upon the reconstitution of the Government it was admittedly necessary that the whole question of pensions should be reconsidered, and the result was the appointment of a Pensions Minister, who quickly got to work and revised the whole scheme.

### Disablement in the Highest Degree.

It is unnecessary to enter into details here with respect to the new pensions arrangements, but as a result of these new arrangements it became essential to modify the provisions of the National Insurance Amendment Act of 1915, previously referred to, in order to give effect to the principle which that Act had intended to confer—namely, relief to the funds of approved societies. This has been done in the new Act by providing, in effect, that when a "war pension" has been granted in respect of "disablement in the highest degree" the insured sailor or soldier is to suffer a reduction in the rate of sickness or disablement benefit to which he would otherwise be entitled under the National Insurance Acts of 5s. a week. The rate of reduction is the same as under the earlier Act, but it is no longer dependent upon the grant of a "total disablement" pension. We have used the words "war pension" to denote a pension granted under any Order in Council relating to pensions of seamen or marines, or of any Royal Warrant relating to pensions of officers or soldiers disabled in consequence of the present war. Reference to the Royal Warrant and Order in Council shows that, among others, the following are to be considered as disablements in the highest degree:—

Loss of two or more limbs.
Loss of an arm and an eye.
Loss of both feet.
Loss of a hand and a foot.
Very severe facial disfigurement.

Now it is clear that such disablement does not necessarily debar the unfortunate sufferer from all possibility of contributing to his own maintenance by undertaking some form of work appropriate to his reduced capacity. The new Act, therefore, goes on to provide that although the reduction in the rate of benefit is to apply throughout the period in respect of which the "war pension" is payable, yet the reduction shall not operate, or shall cease to operate, if the insured person can prove with regard to any claim for sickness benefit that he has been employed, since leaving naval or military service, during twenty-six weeks, whether consecutive or not, and that twenty-six weekly contributions have been paid in respect of him. Similarly, with regard to any claim for disablement benefit the period which will qualify him for restoration to full rate of benefit is 104 weeks, for which weekly contributions have been paid.

### Encouragement to Become Self-supporting.

It is thus apparent that the new legislation contemplates that some efforts will be made to restore the earning capacity of our disabled warriors. The same idea is evinced in another proviso to the first sub-section, which enacts that an allowance in lieu of pension to a person undergoing a special course of medical treatment, or undergoing treatment in an institution, or receiving training in a technical institution, or otherwise, shall be treated as a pension in respect of disablement in the highest degree. Furthermore, it is provided in Section 2 that, when a gratuity or allowance of not less than £30 is made, any part of which is in respect of temporary total disablement, no claim either for sickness or disablement benefit shall be admitted under the National Insurance Acts until the insured person is able to prove that he has been employed, and contributions have been paid, for a total of twenty-six weeks since leaving naval or military service.

These provisions are all in the nature of an encouragement to the pensioner to undertake work suitable to his capacity, and they are, therefore, to be welcomed as designed to assist in the cure of the sufferer, as well as in the best interests of the nation from a purely economical point of view.

The Act has as yet been too short a time in operation to give an opportunity for discovering if the relief which it is to afford to approved societies has become effective. The matter is, however, largely in their own hands, for if they make payments of benefit in full after the date of the commencement of the Act they have no right to recover from the insured persons any sums overpaid, and it therefore behoves approved societies to be on the alert in the examination of all claims for benefit made by discharged sailors or soldiers.

